# Food taboo practices and associated factors among pregnant women in Ethiopia: a systematic review and meta-analysis

**DOI:** 10.1038/s41598-023-30852-0

**Published:** 2023-03-16

**Authors:** Berhanu Gidisa Debela, Daniel Sisay, Habtamu Endashaw Hareru, Helen Ali Ewune, Anene Tesfa, Daniel Alayu Shewaye, Temesgen Muche Ewunie

**Affiliations:** 1 School of Public Health, College of Health and Medical Science, Dilla University, Dilla, Ethiopia; 2 Department of Human Nutrition, College of Health and Medical Science, Dilla University, Dilla, Ethiopia; 3 Ethiopian Public Health Institute, Addis Ababa, Ethiopia

**Keywords:** Health care, Risk factors

## Abstract

Food taboos have a negative impact on pregnant women and their fetuses by preventing them from consuming vital foods. Previous research found that pregnant women avoided certain foods during their pregnancy for a variety of reasons. This review aimed to determine the pooled prevalence of food taboo practices and associated factors in Ethiopia. In compliance with the Preferred Reporting Items for Systematic Reviews and Meta-Analyses (PRISMA) guideline, we searched the literature using PubMed/MEDLINE, AJOL (African Journal Online), HINARI, Science Direct, Google Scholar, and Google electronic databases. The random-effects model was used to estimate the pooled prevalence of food taboo and its determinants at a 95% confidence interval with their respective odds ratios. The pooled food taboo practice among Ethiopian pregnant women was 34.22% (95% CI 25.47–42.96), and after adjustment for publication bias with the trim-and-fill analysis, the pooled food taboo practice of pregnant women was changed to 21.31% (95% CI: 10.85–31.67%). Having less than a secondary education level (OR = 3.57; 95% CI 1.43–8.89), having no ANC follow-up (OR = 4.35; 95% CI 1.12–16.94), and being a rural resident (OR = 3.08; 95% CI 1.14–8.28) were the significant factors. Dairy products, some fruits, green leafy vegetables, meat, and honey are among the taboo foods. The most frequently stated reasons for this taboo practice were: fear of producing a big fetus, which is difficult during delivery; attachment to the fetus's body or head; and fear of fetal abnormality.

## Introduction

During the course of pregnancy, physiological changes like new tissue development and synthesis, mother tissue growth, and the growing fetus significantly increase energy and food needs^1^. To fulfill this increased demand, pregnant women must consume an adequate and balanced diet that includes a sufficient number of macronutrients and micronutrients^2^. Pregnant women restrict themselves from specific foods for several reasons. Pregnant women avoid foods because of a strong dislike (aversion) caused by pregnancy; others avoid them due to medical grounds. However, a high proportion of pregnant women avoid specific foods due to cultural beliefs or impositions in developing countries like Ethiopia^3,4^. Food taboos vary by culture, society, and even among pregnant women, especially in rural areas, and are typically passed down from generation to generation^5,6^.

Although the consumption of important, diversified foods is essential to producing the hormones needed during pregnancy, food taboos during pregnancy are a common practice, particularly in developing countries^7,8^. Prenatal dietary taboo influence what they eat, leaving them vulnerable to micronutrient deficits, including vitamin A, folate, iodine, iron, calcium, and zinc, which are all important during pregnancy. Previous studies from Nigeria^9,10^, Gambia^11^, Kenya^12^, and Ethiopia^13–15^ show that pregnant women are typically prohibited from eating foods high in iron, carbohydrates, animal products, and micronutrients. Avoiding most vital food groups leaves these pregnant women with limited dietary diversity and vulnerable to many nutrient deficiencies, including micronutrient deficiencies, which can lead to pregnancy malnutrition^9,11^. Worldwide, about 9.8 million women are deficient in vitamin A^16^, and iron deficiency, contributes to at least 18% of maternal mortality in developing countries^16,17^. In sub-Saharan Africa, women are particularly exposed to inadequate intake of micronutrients, resulting in different types of malnutrition, which can occur even in the presence of adequate energy and protein intake due to food restrictions during pregnancy^7,18^.

A number of researchers have reported the prevalence of food taboo practices in Ethiopia^15,19–21^. The study done in Addis Ababa shows 18% of pregnant women avoid one or more food items due to food taboos^19^. Previous studies identified age, parity, support from husbands and communities, knowledge, dietary counseling, attendance at antenatal care (ANC), educational status, and cultural beliefs as major determinants^22^. Individual studies on the prevalence of food taboo practice and associated factors in Ethiopia show varying and inconsistent findings. Previous studies conducted on pregnant women's food taboo practices provided a qualitative discussion on the most commonly avoided foods and their reasons, but they didn’t provide pooled food taboo practices among pregnant women in Ethiopia and associated factors using statistical analysis^23^. This systematic review and meta-analysis included the most recent primary studies available, which can provide a recent summarized magnitude of food taboo practices and factors influencing them. By reviewing 16 articles that mentioned food taboos during pregnancy as well as the reasons for the food prohibition, this review may help to fill a gap in summarized information on the prevalence of food taboo practices during pregnancy. The findings of this study can help policymakers, planners, and health service providers by generating and disseminating evidence-based information that can aid in the design and implementation of appropriate interventions. Our study paves the way for future investigations on the determinants of changes and levels of food taboo practices during pregnancy. Hence, the goal of this systematic review and meta-analysis study is to determine the pooled prevalence of food taboos and associated factors among pregnant women in Ethiopia.

## Methods and materials

### Study design and search strategy

We systematically reviewed and analyzed available research articles to determine the pooled prevalence of food taboo practices and associated factors among pregnant women in Ethiopia. The review protocol and reporting were done using the Preferred Reporting Items for Systematic Reviews and Meta-Analyses Protocol (PRISMA-P) and the Meta-Analysis of Observational Studies guideline. The review protocol was filed with the International Registration of Systematic Reviews (PROSPERO) under the registration number CRD42022304915 on February 20, 2022, in order to reduce duplication of reviews and increase transparency in the review process. The study question's core topics were used to develop a search strategy that included food taboo, pregnant women, prevalence, factors, and Ethiopia. For each key concept, appropriate free-text words and Medical Subject Headings (MeSH) were developed. The Boolean logic operators AND, OR, and NOT were used to combine free-text words (truncated or with wildcards) and MeSH terms. The search was not bound by the year of publication, in which case all articles published and/or reported up to December 30, 2022, were included. The electronic search was exhaustively searched by BGD and DAS through PubMed/MEDLINE, AJOL (African Journal Online), HINARI, Science Direct, Google Scholar, and Google electronic databases. In addition, reference lists of already identified articles were also searched to retrieve more relevant studies. The search terms "food taboo" OR "food restriction" OR "food avoidance" OR "food prohibition" AND "prevalence" OR "magnitude" OR "proportion" OR "burden" AND "risk factors" OR "predictors" OR "determinants" AND "pregnant" OR "pregnant woman" OR "pregnant mother" AND "Ethiopia" were used.

### Eligibility criteria and study selection

Cross-sectional, case–control, and cohort studies from the community and facilities were considered. We used both published and unpublished articles in the English language from Ethiopia. Only studies that estimated the proportion and/or associated factors of food taboos were included. Studies that were duplicated, unrelated, or abstract-only were removed, as well as studies with unclear reporting of the burden and/or factors associated with food taboo practices. Two independent reviewers (HEH and DS) acquired the complete texts of the remaining publications and reviewed them thoroughly before data extraction to ensure their eligibility.

### Data extraction procedure and items

Two independent reviewers (BGD and DAS) abstracted data from the primary studies using a standardized data abstraction form devised according to the sequence of variables required. The following characteristics were used to extract data: author's first name, publication date, region; study design (cross-sectional, case–control), sample size, sampling technique, the prevalence of food taboos, odds ratio, and factors that influence food taboos, such as family size, religion, occupation, residence (urban or rural), age, monthly income, education, and ANC follow-up. The degree of agreement between the two independent data extractors is in the range of almost perfect agreement, according to Kappa statistics. We attempted to contact and seek missing outcome data from the original authors by e-mail, and sensitivity analysis was done to find out the robustness of the meta-analysis findings and to show the influence of missing data on review results.

### Quality assessment

The Newcastle–Ottawa Scale for cross-sectional studies was employed to assess the quality of all the included papers for risk of bias^24^. Each paper was critically evaluated by two independent reviewers (BGD and HEH). The problem of subjectivities between the two reviewers was solved through discussion and with the involvement of other review teams (HA, DS, and TME). The papers with a quality evaluation score of 6 out of 10 on the Newcastle–Ottawa Scale were judged low-risk and included in the systematic review and meta-analysis based on the relevant literature cut-off point (Supplementary 2).

### Outcome variable

The primary outcome of this review is the pooled prevalence of food taboo practices among pregnant women at the national level. The numerator was the number of pregnant women who have food taboo practices, while the denominator was the total number of pregnant mothers who participated in the study. The second aim of this review was to identify factors affecting the practice of food taboos among pregnant women in Ethiopia. Unadjusted (raw data) and adjusted odds ratios were extracted for associated factors.

### Statistical analysis

The extracted data was entered into a computer via an Excel sheet for the title, abstract, and full-text screening before being exported to STATA v. 16. The findings were presented in a table and tested using descriptive statistics. The Metaprop program was used to calculate the pooled prevalence of food taboos using the random-effects meta-analysis model. On a forest plot, the single studies and pooled prevalence of food taboo, 95% CI, the author's name, and the publication year were plotted. We performed a subgroup analysis based on the study's publication year, place of residence, and region because the random-effect model with 95% CI had substantial variability among forest plots. Regarding factors influencing food taboo practices, we have pooled the unadjusted odds ratio (raw data) for three factors (age, educational status, and place of residence) (Figs. 6, 7, 9, and 10). However, for ANC follow-up, we pooled both the unadjusted odds ratio (raw data) and the adjusted odds ratio separately, and both were presented to investigate the differences (Figs. 8 and 9). For both adjusted and unadjusted odds ratios, a p-value less than 0.05 with a 95% CI was considered statistically significant. Review Manager 5.4 (RevMan version 5.4)^25^ was used to identify factors influencing food taboo practices among pregnant women using a random-effects model with an inverse-variance method.

### Publication bias and heterogeneity

Heterogeneity between the results of the primary studies was assessed using the Cochran’s Q test and quantified with the inverse variance (I^2^) statistic of 25, 50, and 75% as low, moderate, and severe heterogeneity, respectively, with a p-value less than 0.05. The random-effects model was used to incorporate heterogeneity in meta-analyses. Further, meta-regression was performed using Comprehensive Meta-Analysis software version 4.0^26^ to identify the source of heterogeneity by considering both continuous and categorical data. Region, number of included samples (continuous variable), publication year (during or before 2016 and after 2016), and place of the study (rural/semi-urban, and urban) were considered in the meta-regression. Publication bias was assessed using Begg’s and Egger’s tests with a p-value of less than 0.05 as a cutoff point to declare the presence of publication bias^27,28^. Due to significant publication bias, a non-parametric Duval and Tweedie’s Trim and Fill analysis was undertaken to manage the publication bias^29^ (Fig. 4).

### Ethics approval

No human subject participant was involved.

## Results

### Search results

**Figure 1:**
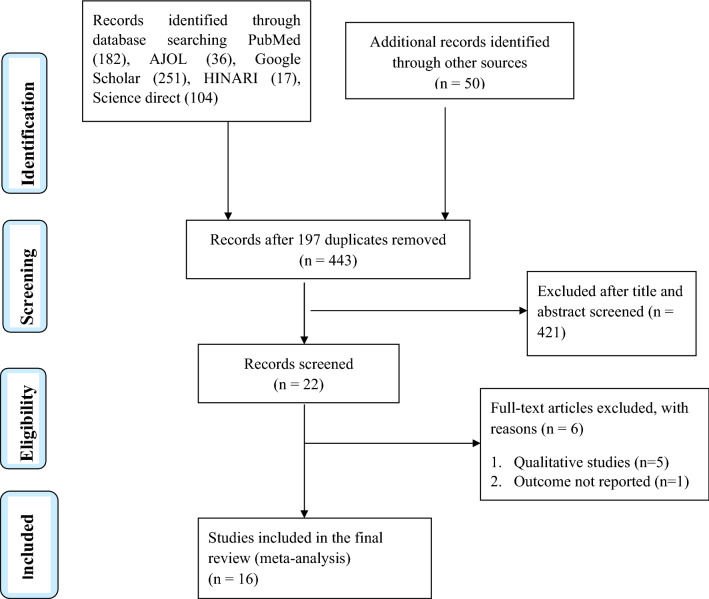
A PRISMA flow chart explaining the selection of primary studies for systematic review and meta-analysis of food taboo practices among pregnant women in Ethiopia.

In the first step of our search, 640 articles were systematically retrieved from electronic databases (PubMed/MEDLINE, Google Scholar, AJOL (African Journal Online), HINARI, Science Direct, and gray literature from Google) and reference lists of previous studies. From the 640 articles, 197 were excluded due to duplication. Additionally, 421 articles were excluded after we reviewed their titles, abstracts, and full texts and found them to be non-relevant to our review. Among the 22 full-text articles accessed, we excluded six articles as they did not report the prevalence of food taboo practices and/or associated factors among pregnant women^15,20,30–33^. Finally, 16 articles were found to be eligible and included in the analysis (Fig. 1).

### Characteristics of included studies

After assessment of the titles, abstracts, and full texts, a total of 16 studies that met the inclusion criteria were included in the review. The included studies were conducted between 1995 and 2022 in the English language. Four studies from each of the Oromia^14,34–36^ and Amhara^37–40^ regional states as well as eight studies from different regions^41–48^ of Ethiopia were included. This review includes five community-based studies and eleven institution-based studies, with sample sizes ranging from 276 to 845 in the Dimma district of Gambella^42^ and West Amhara respectively^39^. A total sample of 6730 pregnant women was included to estimate the pooled prevalence of food taboo practices among pregnant women at the national level. The lowest level of food taboo practice among pregnant women was reported in Mekelle, Tigray region (12%)^41^ and the highest level was reported in Gursum district, Somali region (67.4%)^47^ (Table 1). The quality score of the articles ranged from 6 to 10 out of 10 points (Supplementary 2).

**Table 1:** Summary of included studies on the prevalence of food taboo practices among pregnant women in Ethiopia.

Publication year	First Author	Region	Place	Study design	Sample size	Included	Response rate (%)	Prevalence (95%CI)
2021	Wondimu et al	Oromia	Sendafa Beke	Cross-sectional	422	407	96.45	55.3(50.50–60.13)
2019	Mohammed et al	Addis Abeba	Addis Abeba	Case-control	592	592	100	18.2(15.10–21.31)
2018	Getnet et al	Amhara	Awabel	Cross-sectional	307	300	97.70	27(21.98–32.02)
2015	Zenebe K et al	Amhara	Debretabor	Cross-sectional	355	355	100	45.6(40.42–50.78)
1995	Demissie et al	SNNPRS	Hadiya Zone	Cross-sectional	295	295	100	27(21.93–32.10)
2020	Gebrearegay et al	Tigray	Mekelle city	Cross-sectional	336	332	98.80	12(8.50–15.49)
2015	Zepro N	Oromia	Shashemene	Cross-sectional	295	295	100	49.8(44.09–55.51)
2018	Gedamu et al	Amhara	Meshenti	Cross-sectional	318	318	100	19.5(15.14–23.85)
2015	Tadesse et al	Oromia	limu genet	Cross-sectional	312	303	97.10%	19.1(14.67–23.53)
2021	Ebabu et al	Afar	Aysaita	Cross-sectional	308	308	100%	32.8(27.56–38.04)
2021	Melesse et al	Amhara	West Amhara	Cross-sectional	845	845	100%	19.2(16.54–21.86)
2020	Teshome et al	Gambella	Dimma district	Cross-sectional	276	265	96%	34.7(28.97–40.43)
Unpublished	Ayru A	B/gumuz	Mandura	Cross-sectional	422	422	100%	55.2(50.46–59.94)
2022	Wbalem et al	Oromia	Haramaya	Cross-sectional	422	416	98.6%	48(43.19–52.80)
2022	Tesfa et al	Somali	Gursum district	Cross-sectional	636	610	95.9%	67.4(63.68–71.12)
2022	Melkamsew T. et al	SNNPRS	Southwest zones	Cross-sectional	680	667	98%	17.9(14.99–20.81)

## Meta-analysis

### The pooled prevalence of food taboo among pregnant women in Ethiopia

**Figure 2:**
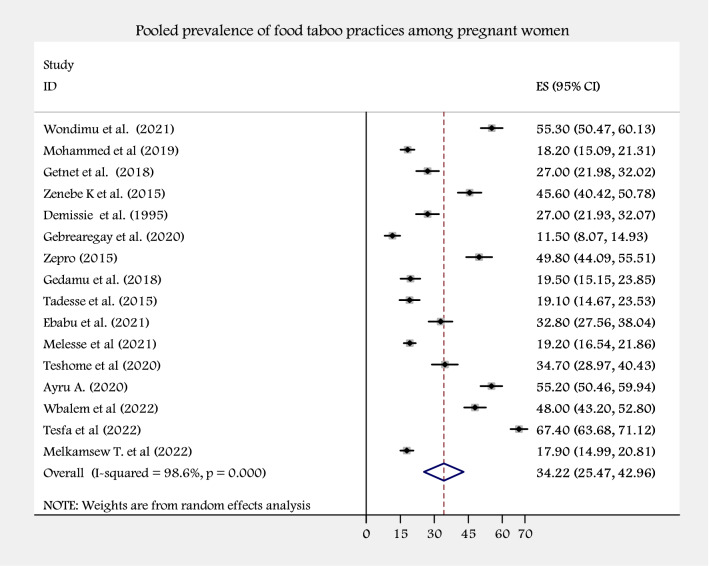
A forest plot of pooled prevalence of food taboo practices among pregnant women in Ethiopia.

The pooled prevalence of food taboo practices among pregnant women in Ethiopia was 34.22% (with 95% CI 25.47–42.96) using visual forest plots in the random-effect model (Fig. 2). Heterogeneity was seen across the studies, which is detected by the I^2^ statistic (I2 = 98.6%, p value < 0.0001). Therefore, we employed a random effect meta-analysis model to estimate the pooled prevalence of food taboo practices among pregnant women in Ethiopia (Fig. 2).

#### Publication bias and Trim-and-fill analysis

**Figure 3:**
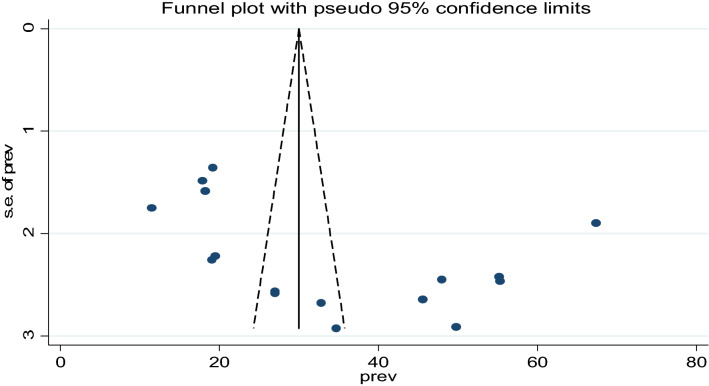
Funnel plot presented the visual inspection of publication bias for systematic review and meta-analysis of food taboo practices among pregnant women in Ethiopia, 2022.

**Figure 4:**
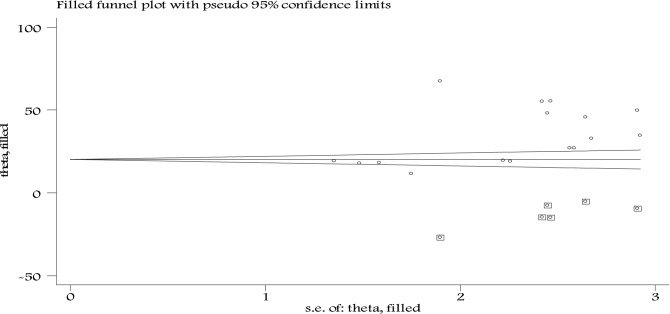
Duval and Tweedie’s Trim and Fill funnel plot.

**Publication bias:** A visual inspection of the funnel plot indicated an asymmetrical distribution of studies showing publication bias (Fig. 3). The presence of publication bias was also checked using Begg's test, which shows insignificant publication bias at a p-value = 0.65, whereas Egger’s test indicates the presence of significant publication bias as evidenced by P = 0.04 (Table 2). Therefore, Duval and Tweedie’s Trim and Fill analysis was done (Fig. 4).

**Table 2:** Egger's test result to assess publication bias.

Std_Eff	Coef	Std. Err	t	P > t	[95% Conf	Interval]
Slope	− 4.586418	15.77879	− 0.29	0.776	− 38.42856	29.25572
Bias	17.22546	7.608603	2.26	0.040	.9066341	33.54429

**Trim-and-fill analysis:** Trim-and-fill analysis shows six studies were imputed for missing studies during the analysis, making the total number of studies of 22 and after adjusting publication bias, the estimated pooled awareness of pregnant women toward food taboo practices appeared to be 21.31% (95% CI 10.85 to 31.67%) (Fig. 4).

### Meta-regression

A meta-regression was conducted to identify the source of heterogeneity using region, residence, sample size, and publication year as covariates. Meta regression revealed that the prevalence of food taboo practices among pregnant women in Ethiopia was significantly associated with the region of the study, meaning that the prevalence of pregnant women food taboo practices was higher among regions categorized as others (Somali, Benishangul Gumuz, Gambela, and Afar) with a coefficient of 1.40, and a p-value of 0.048 when compared with Addis Ababa city administration (Table 3).

**Table 3:** univariate meta-regression analysis result for prevalence of food taboo practices among pregnant women in Ethiopia.

Variables	Categories	Coefficients	P-value
Region	Oromia	1.1789	0.097
	Amhara	0.4944	0.487
	SNNPRs	0.2449	0.753
	Tigray	− 0.5460	0.548
	Others*	1.4017	0.048
	Addis Ababa	REF	
Residence	Rural/semi-urban Urban	0.4930 REF	0.3107
Sample size	Included sample size (continuous)	− 0.0003	0.7970
Publication year	Within and before 2016 After 2016	0.1121 REF	0.8222

Others* = Somali, Benishangul gumuz, Gambela and Afar; REF** = **reference group.

### Sub-group analysis

A subgroup analysis was computed to compare the prevalence of food taboo practices by classifying to region, publication year, and residence place. Based on this analysis, the lowest prevalence of food taboo practices among pregnant women was observed in the Tigray region which is 11.5% (with a 95% CI 8.07–14.93) and the highest prevalence was in the Somali region which is 67.4% (with a 95% CI 63.68–71.12). The pooled prevalence of food taboo practices among the studies conducted after the year 2016 was slightly lower than the pooled prevalence from the studies conducted during and before the year 2016. A higher prevalence of food taboo practices was observed in the studies conducted in rural and semi-urban areas (36.6%) than from those conducted in urban study areas (27.02%) (Table 4).

**Table 4:** Sub-group analysis of food taboo practice among pregnant women in Ethiopia.

Sub group by	Number of studies	Prevalence (95% CI)	P value	I^2^	Tau-squared
Region					
Amhara	4	27.7(16.71–38.69)	0	96.50%	120.85
Oromia	4	43.02 (25.97–60.06)	0	97.90%	296.13
SNNPR	2	22.2(13.30–31.11)	–	89.30%	36.96
Addis Ababa	1	18.2(15.09–21.31)	–	–	0
Tigray	1	11.5(8.07–14.93)	–	–	0
Afar	1	32.8(27.56–38.04)	–	–	0
Gambela	1	34.7(28.97–40.43)	–	–	0
Benishangul Gumuz	1	55.2(50.46–59.94)	–	–	0
Somali	1	67.4(63.68–71.12)			0
Time of study					
After year 2016	12	33.89(23.30–44.49)	0	98.90%	345.8
Within and before 2016	4	35.3(20.75–49.87)	0	97.00%	213.9
Place residence					
Urban	4	27.02(13.45–40.59)	0	97.70%	186.9
Rural or semi urban	12	36.6(25.96–47.33)	0	98.70%	351.3

### Sensitivity analysis

**Figure 5:**
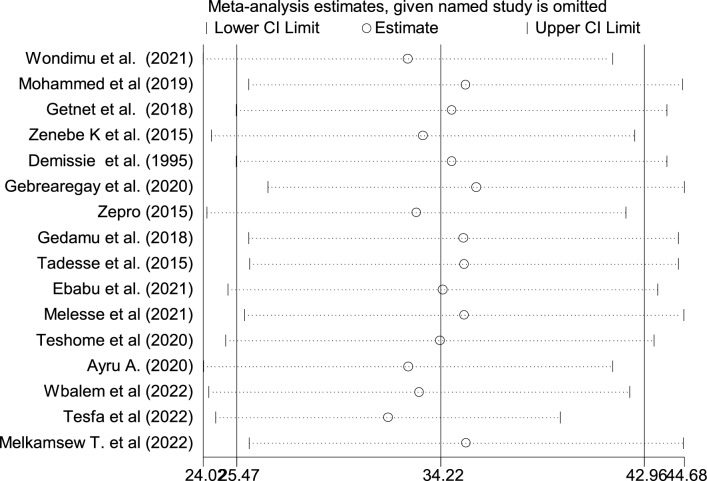
Sensitivity analysis of the pooled prevalence of included studies (n = 16).

A sensitivity analysis was done to evaluate the effect of a single study on the overall effect estimate. The results indicated that removing a single study did not have a significant influence on pooled prevalence (Fig. 5).

### Factors associated with pregnant women's food taboo practices in Ethiopia

**Figure 6:**
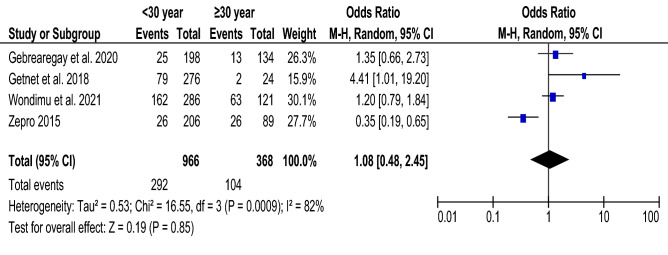
Forest plot using an unadjusted odds ratio for the association between age and food taboo practices among pregnant women in Ethiopia, 2022.

**Figure 7:**
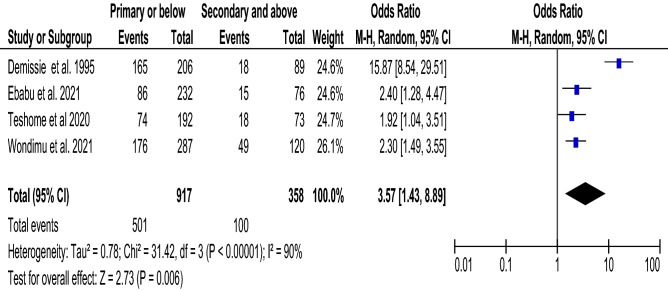
Forest plot using an unadjusted odds ratio for the association between education and food taboo practices among pregnant women in Ethiopia, 2022.

**Figure 8:**
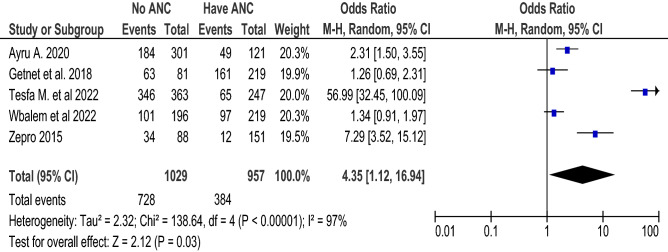
An unadjusted odds ratio forest plot for the Association of ANC with food taboo practices among pregnant women in Ethiopia, 2022.

**Figure 9:**
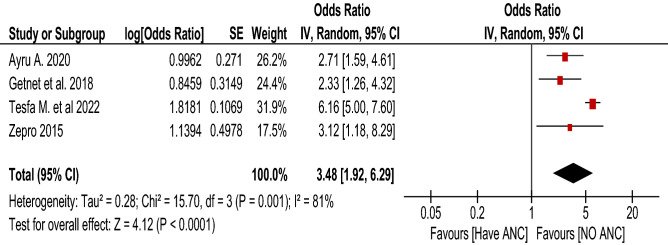
Forest plot using adjusted odds ratio for the association of ANC with food taboo practices among pregnant woman in Ethiopia, 2022.

**Figure 10:**
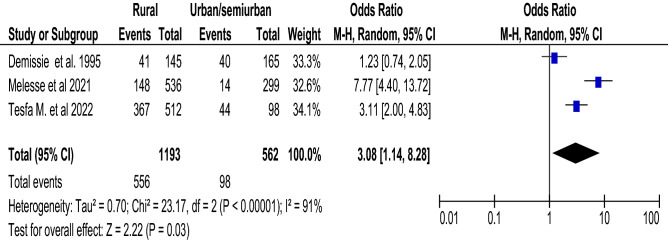
Association between place of residence and food taboo practices among pregnant women in Ethiopia, 2022**.**

Eleven studies^14,34,36,37,41–44,46,47^ were included for the analysis of risk factors for food taboo practices. Accordingly, four risk factors had data that could be used in the quantitative meta-analysis. The pooled odds ratios of four identified risk factors were ranged from 1.08 (for age) to 4.35 (for ANC follow-up) ((Figs. 6, 7, 8, 9, 10).

### Age and food taboo practices

A total of four articles^14,34,37,41^, with 1334 participants were included to assess the association between age and food taboo practices. The pooled odds ratio of included studies shows there is no significant association between the age of pregnant women and food taboo practices (Fig. 6).

### Educational status and food taboo practices

Four articles^34,42,43,46^ with a total of 1275 participants were included in the analysis to detect the association between education and food taboo. Pregnant women with primary and below educational status were 3.57 times more likely (with 95% CI 1.43, 8.89, and I^2^ = 90%) to practice food taboos during pregnancy than those with secondary and above educational status (Fig. 7).

### ANC counseling and food taboo practices

A total of five articles^36,37,39,41,44^ with 1986 participants were included to determine the association between ANC counseling and food taboo practices using an unadjusted odds ratio. Those mothers who had no ANC follow-up were 4.35 times (with 95% CI 1.12, 16.94, I^2^ = 97%) more likely to practice food taboo as compared to those who had ANC follow-up (Fig. 8). The pooled AOR (Fig. 9) was 3.48 (with 95% CI 1.92, 6.29; I^2^ = 81%; n = 4 studies). Both the unadjusted OR and AOR suggest that pregnant women who have no ANC follow-up have significantly higher odds of engaging in taboo food practices when compared to those who have ANC follow-up during their pregnancy.

### Place of residence and food taboo practices

Three articles^39,46,47^ with a total of 1755 participants were included in this analysis. Mothers who live in rural areas were 3.08 times (with 95% CI 1.14, 8.28; I^2^ = 91%) more likely to practice food taboo during their pregnancy than those who live in urban areas (Fig. 10).

### Types of foods considered taboo in Ethiopia and the reasons

Among the 16 included studies, 13 of them assessed the types of prohibited foods for pregnant women and their reasons. From the findings of the studies, seven of them^14,34,38,41,43,44,46,48^ reported that mainly milk and milk products were prohibited due to beliefs that they plastered on the fetal head, prolonged labor, fear of abortion, making the baby fat, disclosures of the fetus, etc. Fruits and vegetables were also among the most frequently reported food types prohibited by pregnant women due to the most common reasons: attachment to the fetus's body, making a fatty baby, and fear of fetal abnormality. Other types of prohibited foods were meat and eggs due to the fear of producing a large fetus, which is difficult to deliver (Table 5).

**Table 5:** Foods considered taboo in Ethiopia and the reasons for their prohibition among pregnant women.

First Author	Region	Foods that are considered taboo	Major reasons for food taboos or prohibitions
Wondimu et al.^34^	Oromia, Sendafa Beke	Milk and milk products, eggs, linseed, fatty meats, fruits, honey, and vegetables	Plastered on the fetal head Fear of abortion, and Fatty baby
Mohammed et al^45^	Addis Abeba	Green chili pepper, organ meat, and dark green leafy vegetables like spinach, lettuce, kale, and broccoli	Traditionally held myths and misinformation
Getnet et al.^37^	Amhara Awabel	Linseed, coffee, tea, cabbage, porridge, wheat bread, banana, pimento, groundnut, salty diet, nug, sugarcane, pumpkin, and Coca-Cola drinks	Plastered on the foetal head, fatty baby, abortion fear, and Fetal abnormality
Demissie et al.^46^	SNNPRS Hadiya Zone	Milk and cheese, linseed, and fatty meat	Fear of difficult delivery, discoloration of the fetus, and fear of abortion
Gebrearegay et al.^41^	Tigray Mekelle city	Yogurt and milk, banana, legumes, honey, and "kollo" (roasted barley and wheat), mustard, porridge	cause abortion, abdominal cramps in the mother and newborn, prolonged labor, or coating of the fetus’s body
Zepro N.^14^	Oromia Shashemene district	Linseed, honey, milk, fatty meat, eggs, fruits, and vegetables	Plastered on the fetal head, it causes a fatty baby and a difficult delivery, as well as abortion fear and fetal abnormality
Gedamu et al.^38^	Amhara Meshenti	Vegetables, meat, paper, pourage, sugar cane, and yogurt	Plaster the fetal head, make the baby fat, and make delivery difficult
Ebabu et al.^43^	Afar Aysaita	Milk, honey, fruits, fafa, and vegetables	Fear of a difficult delivery, fetal disclosures, Fear of abortion
Teshome et al.^42^	Gambella, Dimma district	Fruits, cereals, honey, sugarcane, garden cress, mustard seed, and yam	Fear of maternal and fetal complications, fear of abortion, cardiac problems, and anemia
Ayru A.^44^	Benishangul gumuz, Mandura	Milk/yoghurt, egg, fatty meat, honey, fruits, and vegetables	Nausea, abortion, difficulty during delivery—plastered in the chilled head
Wbalem et al.^36^	Oromia, Haramaya district	Meat, salt, egg, cabbage, milk, and oil	Fear of difficult delivery, fear of rising blood pressure, fear of protracted labor, and fear of fetal body deformities
Tesfa et al.^47^	Somali, Gursum district	Meat, egg, carbonated drinks, pasta with sauce and milk	Fearing a difficult delivery, Fear of abortion and fear related to plaster the fetal head

## Discussion

Ethiopian pregnant women avoid certain foods for a range of reasons, and some of these relate to factors associated with pregnancy outcome, the birthing process, culture, and others to avoid undesirable aesthetic features in the baby^14,15^. This study, the first systematic review and meta-analysis as far as our knowledge goes, is aimed at estimating the pooled prevalence of food taboo practice and associated factors among pregnant women in Ethiopia. The findings of this meta-analysis showed that the pooled proportion of food taboo practices among pregnant women in Ethiopia is 34.22% (95% CI 25.47–42.96), and after adjustment for publication bias with the trim-and-fill analysis, the pooled food taboo practice of pregnant women was changed to 21.31% (95% CI: 10.85–31.67%). Since we were unable to find another meta-analysis that reported food taboo practices among pregnant women, we didn't compare the findings with similar studies.

The subgroup analysis shows a variation of food taboo practices between regions, with the lowest (11.5%; 95% CI 8.50–15.49) from the Tigray region and the highest (67.4%; 95% CI 63.68–71.12) from the Somali region. The variation could be due to the difference in health service accessibility and utilization. This possible explanation is supported by evidence from the 2019 Ethiopian Mini-Demographic Health Survey (EDHS 2019)^49^ and recent reports^50^ on "Progress in Health among Regions of Ethiopia", show regional differences in health service accessibility and utilization. With regard to variation in health service utilization among regions the data from the 2019 Ethiopian Mini-Demographic Health Survey (EDHS 2019)^49^ show the percentage of pregnant women who received antenatal care from a skilled provider was 70.8% in Oromia and 30.2% in Somali region.

The variation could also be due to the difference in educational status of study participants, which varies among regions. According to the Ethiopian Demographic Health Survey (EDHS 2016)^51^ the percentage of the female household population who completed primary education ranges from 5.2% in Addis Ababa city administration to 0.7% in the Somali region, which may influence their level of food taboo practices. Furthermore, the possible explanation could be due to socio-cultural differences, as food taboo practices are strongly linked to specific communities' cultural beliefs, as the Federal Democratic Republic of Ethiopia is divided into ethnic-linguistic regions.

The pooled prevalence of food taboo practices among the studies conducted after the year 2016 was slightly lower than the pooled prevalence from the studies conducted during and before the year 2016, which was 33.89% and 35.3%, respectively. The possible explanation for the difference could be an improvement in the accessibility of health services, which increases community awareness, particularly through Ethiopian health extension workers. Another reason could be the improvement of the educational levels of women and their partners, as the accessibility of education also shows improvement from time to time.

The findings of this review show the most commonly taboo food types are from four major food groups: proteins, fats, and fruits and vegetables. These include green chili pepper, spinach, lettuce, kale, broccoli, linseed, cabbage, banana, pimento, groundnut, sugarcane, pumpkin, fatty meat, egg, honey, and some cereal products. This is similar to a meta-analysis conducted on food taboos^52^ and a narrative literature review done in Ethiopia^53^, which show similar types of foods were prohibited by pregnant women. Among the reasons explained for food taboo, foods getting attached to the fetus's body or head, making the baby gain weight, and fear of fetal abnormality are the most frequent ones. This is similar to a literature review done in Ethiopia^53^, a meta-analysis conducted worldwide^52^, and a systematic review conducted in Ethiopia^23^, which show fear of producing a big fetus, which is difficult during delivery or prolonged labor, fear of abortion, discoloring of the fetus, nausea, abdominal cramps in the mother, and fetal head plastering as the primary drivers of food taboo. Another systematic review supports this, finding that pregnant women's food restrictions are related to their fear of unfavorable pregnancy outcomes, such as the risk of abortion, and are used to avoid child problems such as cutaneous and respiratory disorders^54^.

Regarding factors associated with food taboo practices, the odds of practicing food taboo during pregnancy were 3.57 times (OR = 3.57; 95% CI 1.43–8.89) higher among pregnant women who have primary and below educational status when compared with those who have secondary and above educational status. This finding is supported by meta-analysis study^55^ from Ethiopia, which found that pregnant women with lower educational levels have less dietary diversity than those with higher education levels, possibly due to food taboos. This finding also aligned with other primary studies carried out in Sudan and Nigeria, which indicated that higher maternal education was linked to a lower likelihood of practicing food taboos during pregnancy^56,57^. The possible reason could be the knowledge that they obtained from formal education and reading from different sources about appropriate maternal nutrition.

The ANC follow-up of pregnant women was also significantly associated with food taboo practices during pregnancy. The finding shows pregnant women who had no ANC counseling were 3.48 (AOR = 3.48; 95% CI 1.92–6.92) times more likely to practice food taboos during the pregnancy period than those who had ANC counseling. The finding is in line with a review of evidence on traditional beliefs and practices during the pregnancy period in Asian countries, which suggests that nutrition education should be given to both mothers and husbands in order to alleviate malpractices during pregnancy^58^. The possible reasons for the association may be due to the nutrition counseling provided by health care providers’ during ANC follow-up.

Another factor that significantly affected the pregnant woman's food taboo practices was her place of residence. Pregnant women who reside in rural areas are 3.08 (OR = 3.08; CI 1.14–8.28) times more likely to practice food taboo than those who live in urban areas. The finding is slightly supported by the meta-analysis study from Ethiopia, which shows pregnant women who reside in rural areas are more likely to get inadequate dietary diversity than those who live in urban^55^. The probable justification is that urban mothers have more access to health services and health information from different sources than rural pregnant women.

## Conclusion

According to the results of this meta-analysis, more than one-third of pregnant Ethiopian women practice food taboo during their pregnancy. Dairy products, fruits, vegetables, meat, eggs, and honey were among the foods prohibited for pregnant women. Food clinging to the fetus's body or head, fear of having a huge fetus that would be difficult to deliver, fear of abortion, and fear of the fetus's appearance were the most often reported reasons for food taboo practices. Educational status, ANC follow-up, and place of residence had a substantial impact on the outcome variable. Therefore, healthcare organizations and other concerned bodies should work on reducing food taboo practices focusing on the identified factors. The findings can be used by healthcare professionals, managers, and policymakers to improve nutrition programs by devising plans and making active efforts to address gaps in dietary practices during pregnancy.

### Strength and limitation of the study

We strictly followed PRISMA flow charts and searched a wide range of databases to retrieve related articles. To provide a comprehensive review, additional searches such as hand searching reference lists and citation searching were conducted. Procedures to minimize human error and subjectivity like double independent screening, and double independent quality assessment was used. The limitation of this systematic review and meta-analysis might be due to the cross-sectional nature of the included studies, which couldn’t show the temporal relationship between the outcome and independent variables. Another limitation might be due to the presence of heterogeneity between studies and publication bias, which readers should consider when using this finding. We also have faced difficulties comparing our findings with other findings due to the lack of similar systematic reviews and meta-analyses.

## Supplementary Information

Supplementary Information 1.

Supplementary Information 2.

Supplementary Information 3.

## Data Availability

All data generated or analyzed during this study are included in this published article (and its Supplementary Information files).
