# Supplementary material for: Food taboo practices and associated factors among pregnant women in Ethiopia: a systematic review and meta-analysis
Source: Sci Rep. 2023 Mar 16;13:4376. doi: 10.1038/s41598-023-30852-0 (PMC10020167; doi:10.1038/s41598-023-30852-0)
Supplement: Supplementary file 3 — Supplementary Information 3. [file 41598_2023_30852_MOESM3_ESM.docx]

Food taboo practices and associated factors among pregnant women in Ethiopia: A systematic review and meta- analysis

Berhanu Gidisa Debela^1^*, Daniel Sisay^1^, Habtamu Endashaw Hareru^1^, Helen Ali Ewune^2^, Anene Tesfa^3^, Daniel Alayu Shewaye^1^, Temesgen Muche Ewunie^2^

^1^School of Public Health, College of Health and Medical Science, Dilla University, Ethiopia

^2^Department of Human Nutrition, College of Health and Medical Science, Dilla University, Ethiopia; ^3^Ethiopian Public Health institute

*Corresponding Author: [berhanugdebela@gmail.com](mailto:berhanugdebela@gmail.com)(BGD)

(Supplementary2). Quality assessment of studies using the modified Newcastle Ottawa scale for cross sectional studies for systematic review meta-analysis of prevalence and determinants of food taboos among pregnant mothers in Ethiopia: Systematic review and Meta analysis.

|  | **Selection ( 5 stars)** | | | | **Comparability ( 2 stars)** | **Outcome ( 3 stars)** | | Total quality  score  (**10*)** |
| --- | --- | --- | --- | --- | --- | --- | --- | --- |
| **Study ID** | Representativeness of the sample(*) | Samples size(*) | Non- respondents(*) | Ascertainment of the exposure(**) | Confounding factors controlled(**) | Assessment of outcome(**) | Statistical test(*) |  |
| Wondimu *et al*. | * | * | * | ** | * | ** | * | *********(9) |
| Mohammed et al | * | * | * | * | ** | ** | * | **********(10) |
| Getnet et al. | * | * | * | * | ** | * | * | ********(8) |
| Zenebe K et al. | * | * | * | * | * | * | * | *******(7) |
| Demissie et al. | * | * | * | ** | - | * | - | *******(6) |
| Gebrearegay et al. | * | * | * | * | ** | * | * | ********(8) |
| Zepro | * | * | * | ** | * | * | * | ********(8) |
| Gedamu et al. | * | * | * | ** | - | * | - | ******(6) |
| Tadesse et al. | * | * | * | * | - | * | * | ******(6) |
| Ebabu et al. | * | * | * | * | * | * | * | *******(7) |
| Melesse et al | * | * | * | * | ** | * | * | ********(8) |
| [Teshome et al](https://www.magonlinelibrary.com/doi/abs/10.12968/ajmw.2020.0006) | * | * | * | * | ** | * | * | ********(8) |
| Ayru A. | * | * | * | ** | ** | ** | * | *********(10) |
| Wbalem et al | * | * | * | ** | ** | ** | * | *********(10) |
| Tesfa M. et al | * | * | * | ** | ** | ** | * | *********(10) |
| Melkamsew T. et al | * | * | * | ** | * | ** | * | *********(9) |
